# Public health implications of contamination of Franc CFA (XAF) circulating in Buea (Cameroon) with drug resistant pathogens

**DOI:** 10.1186/1756-0500-7-16

**Published:** 2014-01-08

**Authors:** Jane-Francis Tatah Kihla Akoachere, Nana Gaelle, Henry Meriki Dilonga, Theresa K Nkuo-Akenji

**Affiliations:** 1 Department of Microbiology and Parasitology, Faculty of Science, University of Buea, Buea, Cameroon; 2 Laboratory for Emerging Infectious Diseases, Faculty of Science, University of Buea, Buea, Cameroon

**Keywords:** Currency, Franc CFA (XAF), Contamination, Pathogens, Antibiotics, Susceptibility, Resistance, Bacteria, Fungi, Cameroon

## Abstract

**Background:**

Studies in different parts of the world have implicated money as a vehicle for transmission of pathogens. Such information which is necessary to facilitate infection control strategies is lacking in many sub-Saharan countries including Cameroon. This study analyzed the Franc de la Communauté Financiere d’Afrique (Franc CFA), the currency used in Cameroon and other countries in the Central African sub-region, as a potential vehicle for transmission of pathogenic bacteria and fungi, particularly drug-resistant strains, to generate findings which could create awareness on currency contamination and serve as a guide when formulating health policies on currency.

**Methods:**

Two hundred and thirteen currency samples representing various denominations of notes and coins randomly collected from diverse sources in Buea, Cameroon were analyzed for bacteria and fungi. The sensitivity of bacterial isolates to antibiotics was tested using the disc diffusion method. The relationship between contamination and physical state, source or denomination of currency was assessed using the χ^2^ test. All statistics were discussed at 0.05 significance level.

**Results:**

Two hundred (93.9%) samples were contaminated with notes (96.6%) showing higher contamination than coins (88.2%). Uncirculated (mint) samples showed no contamination. There was a significant difference (P˂0.05) in contamination with respect to currency denomination, physical state and source. All samples from butchers and patients/personnel in hospitals were contaminated. Lower denominations showed significantly higher (P = 0.008) levels of contamination than higher denominations. Dirty currency was more contaminated than clean currency. Nine bacterial species were isolated. Coagulase-negative *Staphylococcus* (CoNS) (54.9%) and *Staphylococcus aureus* (20.1%) predominated. Among the fungi detected, *Aspergillus sp* (17.3%) and *Penicillium sp* (15.9%) showed higher frequency of occurrence. Bacteria were susceptible (100%) to ceftriaxone, gentamicin, norfloxacin and ofloxacin. Susceptibility to amoxicillin, penicillin, ampicillin, vancomycin and cotrimoxazole was low. Staphylococci were resistant (100%) to vancomycin, penicillin G, and amoxicillin. CoNS in addition showed resistance (100%) to cotrimoxazole.

**Conclusions:**

The CFA franc circulating in Buea could serve as a vehicle for transmission of drug resistant pathogenic or potential organisms and contamination could be due to currency usage and handling as mint notes were not contaminated. Hygiene practices during or after handling currency is greatly encouraged to prevent infection.

## Background

A classic characteristic of microbial agents is the evolution of routes for their transmission to susceptible hosts [1]. These routes of transmission are of great importance in the health of many populations particularly in developing countries, where the frequency of infection is a general indication of local standards of hygiene and environmental sanitation [2]. Currency is an exchangeable fomite continuously subjected to contamination by various substances ranging from illicit drugs [3-5] to microorganisms [6]. Communicable diseases can spread through contacts with contaminated fomites, hence, currency could play a role and thus presents a public health threat. The possibility that currency notes and coins could act as environmental vehicles for the transmission of potential pathogenic microorganisms was suggested as early as in the 1970s by Abrams and Waterman [7] particularly as money is frequently transferred from one person to another [8]. Because facts to support this are not sufficient, the World Health Organization [9] therefore recommends that more studies be carried out in other locations particularly Africa to evaluate the public health implications of microorganisms on money.

At present, there are very few investigations on currency contamination by microorganisms. Most of the studies carried out so far are in developed countries [10-12], findings of which have created a high level of awareness on the potential for currency contamination by microorganisms and hence it’s potential as a vehicle for transmission of pathogens. These studies have also resulted in the creation of national agencies charged with handling of mutilated currency. Scientific data on microbial currency contamination in developing countries is inadequate. This dearth of information may have contributed to the absence of public health policies on currency usage, handling and circulation in developing countries. Although there are some reports on bacteriological examination of paper currency [13,14] very few studies have analyzed contamination by fungi. In addition, very few reports exist on the microbial examination of monetary coinage.

In sub-Saharan Africa, few studies carried out have investigated microbial contamination of currencies such as the Nigerian Naira [15-18], the South African Rand [19] and the Ghanian Cedi [20]. Very few of these studies have investigated fungal contamination and antibiotic sensitivity of contaminating microbial species. Considering the fact that resistance plasmids as well as virulence markers have been detected in isolates from currency [18,21] implying that currency may not only serve as a vehicle for transmission of pathogenic bacteria but also a reservoir for resistance plasmids which could be transferred to susceptible organisms worsening drug-resistance burden, studies on antibiotic susceptibility of pathogens from currency are necessary to determine the appropriate antibiotics to be employed in the effective treatment of infections for which contaminated currency is implicated [22]. Since no study has been carried out evaluating the potential of the Franc de la Communauté Financiere d’Afrique (Franc CFA) (XAF) there is thus no information on its potential in transmission of pathogens. The Franc CFA (XAF) is the currency used by six countries in the Central African sub-region: Cameroon, Chad, Central African Republic, Republic of Congo, Equatorial Guinea and Gabon which form the Central African Economic and Monetary Community (CEMAC). This lack of data on contamination may have contributed to the absence of public health policies on the usage and handling of the Franc CFA (XAF). Our study, first of its kind, analyzed the Franc CFA (XAF) as a potential vehicle for transmission of pathogenic bacteria and fungi, particularly drug-resistant strains. Findings of this study will have far-reaching public health implications and will serve as basis for health consciousness during currency handling, for effective control of transmission of infection as well as a guide when formulating health policies on currency.

## Methods

### Study site

The study was carried out in Buea, the headquarters of the South West region, Cameroon. The town of Buea has a multiethnic population and is situated in the equatorial forest zone just by the foot of Mount Cameroon. This area has a rainy season running from March to November and a dry from December to mid-March [23]. Like any other part of Cameroon, Buea has its own economic activities thus goods and services are exchanged for money between members of this community. The location of the University of Buea and other educational institutions in Buea have brought about an overwhelming increase in population and with this increase there is a corresponding rise in the number of financial institutions and businesses of all magnitudes. This has increased the level of business transactions with other communities out of Buea. There is therefore intense circulation of money in Buea.

### Study design

This study was based on laboratory investigations on various denominations of currency randomly collected from predetermined categories of persons and institutions in Buea from April to September 2011. Money was collected only from individuals who accepted to participate freely in the study. A verbal authorization was received from the Directors of participating hospitals (Regional Hospital Annex, Buea and Solidarity Clinic Molyko) and the bank, Banque Internationale du Cameroun pour l’Eparnge et le Credit (BICEC), in response to a request for administrative authorization to collect samples from cashiers in these institutions.

### Sample collection

Eight denominations of Franc CFA (XAF) notes and coins were randomly collected from various categories of persons and institutions namely: butchers, food vendors (fish/meat roasters, restaurants, and snack corners), students/pupils, taxi drivers, store attendants and hospital/clinic cashiers. The different denominations were used in purchasing goods and services from subjects within these predetermined categories so as to obtain change. It was by this method that we obtained a variety of currency samples. The change so returned was collected in sterile polyethylene bags and sealed to avoid further contamination. Mint currency was collected from BICEC. Samples were labelled, transported to the laboratory and then processed immediately for the isolation and identification of microorganisms.

### Physical condition of currency

The physical state of currency was assessed and categorized as mint, clean, or dirty/mutilated. The term ^’^mint^‘^ describes uncirculated currency notes that had been newly produced. Such currency had not gone into circulation as such was used as control in this study. ‘Clean^’^ describes notes/coins that were not dirty and damaged. This included currency in the grades specified by the International Bank Society [24] as extremely fine, very fine, fine, very good and good. ^‘^Dirty^’^/‘mutilated^’^ currency describes notes/coins that were dirty, damaged, soiled, smelly, molded or held together with sticky tape. This was currency in the grades fair and poor.

### Isolation and identification of microorganisms

A sterile cotton swab moistened with sterile physiological saline (0.85% NaCl) was used to swab both sides of each note or coin. To isolate bacteria, the swab was used to inoculate Blood agar, McConkey agar, Eosine Methylene Blue agar and Mannitol Salt agar plates by streaking. The plates were then incubated aerobically at 35-37°C for 24–48 hours to allow organisms to grow. Pure cultures were obtained by streaking a small portion of cells from an isolated colony on the corresponding medium and incubating plates over night at 35°C-37°C. Colonial morphology as well as haemolytic reactions was observed. Pure cultures were Gram-stained, tested for motility and further characterized using standard biochemical techniques. Enterobacteriaceae were further characterized using the API 20E kit (Analytical Profile Index, BioMerieux, Durham, NC, USA).

To isolate fungi, Sabouraud Dextrose agar supplemented with 0.01% chloramphenicol was inoculated with swab by streaking. Plates were inoculated in duplicates. One plate was incubated aerobically at 37°C for 48 hours to permit the growth of pathogenic fungi. The second plate was incubated for five days at room temperature to isolate the mold. Molds were identified on the basis of cultural (pigment production, if in mold or yeast form) and microscopic morphological characteristics following staining with lactophenol cotton blue. Yeast on the other hand was identified on the basis of colour (pigmentation), cellular morphology (following gram staining) and where applicable, by biochemical tests.

### Antibiotic susceptibility testing of bacterial isolates

Bacteria were tested for susceptibility to antibiotics by the Kirby-Bauer disc diffusion technique following guidelines of the National Committee for Clinical and Laboratory Standards Institute [25]. Fourteen different antibiotic discs (from Liofilchem S.R.L., Italy) representing antibiotics commonly used in the treatment of infections in study area were tested. Antibiotics tested were Amoxicillin (AML, 30 μg), Ampicilin (AMP, 10 μg), Cefotaxime (CTX, 30 μg), Ceftriaxone (CRO, 30 μg), Cefuroxime (CXM, 30 μg), Chloramphenicol (C, 30 μg), Cloxacillin (CX, 5 μg), Cotrimoxazole (SXT, 25 μg), Erythromycin (E, 15 μg), Gentamicin (CN, 10 μg), Oxacillin (OX, 1 μg), Penicillin G (P, 10 μg), Tetracycline (TE, 30 μg) and Vancomycin (VA, 30 μg). Susceptibility assay was performed on Mueller–Hinton agar plates. Briefly a suspension of the test organism was prepared to match the 0.5% McFarland standard. Plates were swabbed with the cell suspension and allowed to dry for 10 minutes. Different antimicrobial discs were placed on the inoculated plates ensuring adequate contact of disc and medium. Plates were incubated at 37°C for 24 hours, examined and the diameter of the zone of inhibition measured using a graduated ruler. The diameters were compared with recommended standards, which conform to those of the Clinical Laboratory Standard Institute [25].

### Data analysis

Data was entered in EPI Info 6.04d (CDC, 2001) and exported to the software package, SPSS version 17.0 (IBM-SPSS 2008) for analysis. Descriptive statistics were performed using frequencies and proportions. Multiple counting was computed using multiple response analysis (MRS). Association or relationship between indicators was assessed using the Chi-square test (χ^2^-test) of independence. All statistics were discussed at the 0.05 significance level (alpha ≤0.05), that is a p-value less than 0.05 was said to be significant.

## Results

### Sample description

A total of 213 money samples comprising 500 frs (22, 10.3%), 1000 frs (49, 23.0%), 2000 frs (44, 20.7%), 5000 frs (15, 7.0%), 10000 frs (15, 7.0%) as notes and 500 frs (9, 4.2%), 100 frs (37, 17.4%) and 50 frs (22, 10.3%) as coins collected from various sources were investigated (Additional file 1). There was a higher frequency of circulation for the 2000 frs notes, 1000 frs notes and 100 frs coins when compared with the other currency denominations sampled. Sixty five (30.52%) samples (i.e. 35 notes and 30 coins) were ‘clean’ while 148 (110 notes and 38 coins) (69.48%) were ‘dirty’. The dirtiest were found among the 1000 frs notes (38, 17.8%), 2000 frs notes (34, 15.9%) and 100 frs coins (24, 11.35) (Additional file 1).

### Relationship between contamination and denomination, physical appearance and source of currency

A total of 200 (93.8%) currency samples were contaminated. Generally notes (140/145, 96.6%) showed higher contamination than coins (60/68, 88.2%) with the 500 frs notes (100%) showing highest level of contamination (Table 1). Forty-six (21.6%) notes and 27 (12.6%) coins were contaminated solely with bacteria, 7 notes (3.3%) and 9 (4.2%) coins were contaminated solely with fungi while 87 (40.8%) notes and 24 (11.3%) coins were contaminated with both bacteria and fungi (mixed contamination) (Table 1). There was a significant difference in microbial colonization of currency with respect to denomination (χ^2^ = 7.034, P = 0.008, df = 1 for bacteria; χ^2^ = 5.320, df = 1, P = 0.021 for fungi; χ^2^ = 6.241, df = 1, P = 0.018 for mixed contamination) with lower denominations (500 frs, 1000 frs, 2000 frs notes and 50 frs coins) showing higher levels of contamination than higher denominations (Table 1). Microorganisms were not recovered from the 16 mint samples.

**Table 1:** Relationship between currency denomination and type of contamination

**Currency denomination (N)**		**Contamination N (%)**			**Total number contaminated N (%)**
		**Strictly bacteria**	**Strictly fungi**	**Bacteria and fungi**	
**Note**	500 (22)	9 (4.2)	0 (0)	13 (6.1)	22 (100)
	1000 (49)	13 (6.1)	2 (0.9)	32 (15.0)	47 (95.9)
	2000 (44)	12 (5.6)	3 (1.4)	28 (13.1)	43 (97.7)
	5000 (15 )	9 (4.2)	1 (0.5)	4 (1.9)	14 (93.3)
	10000 (15)	3 (1.4)	1 (0.5)	10 (4.7)	14 (93.3)
	**Total (145)**	**46 (21.5)**	**7 (3.3)**	**87 (40.8)**	**140 (96.6)**
**Coin**	50 (22)	11 (5.2)	1 (0.5)	8 (3.7)	20 (90.9)
	100 (37)	14 (6.6)	6 (2.8)	12 (5.6)	32 (86.5)
	500 (09)	2 (0.9)	2 (0.9)	4 (1.9)	8 (88.9)
	**Total (68)**	**27 (12.7)**	**9 (4.2)**	**24 (11.3)**	**60 (88.2)**
**Grand total (213)**		**73 (34.2)**	**16 (7.5)**	**111 (52.1)**	**200 (93.6)**

Bacteria: χ^2^-test: χ^2^ = 7.034; df =1; P = 0.008; Fungi: χ^2^-test: χ^2^ = 5.320; df =1; P = 0.021.

Bacteria and fungi: χ^2^-test: χ^2^ = 6.241; df =1; P = 0.018; Currency denomination: χ^2^-test: χ^2=^ 3.868; df = 1; P = 0.049; N = Number obtained.

Contamination with either bacteria alone, fungi alone or mixed bacteria and fungi was generally significantly higher in dirty currency than in clean currency (Table 2). Comparing the prevalence of bacterial contamination on clean and dirty currency, there was a significant difference observed with notes (χ^2^ = 13.29, df = 1, P = 0.001) but not with coins (Table 2). There was no significant difference observed in fungal contamination between clean and dirty notes (χ^2^ = 0.00, df = 1, P = 0.991) and between clean and dirty coins (χ^2^ = 0.003, df = 1, P = 0.959). Mixed contamination was higher in notes (40.8%) than in coins (11.2%).

**Table 2:** Physical appearance of currency and type of contamination

**Currency physical appearance**		**Contamination N (%)**			**Total number contaminated ** **N (%)**
		**Strictly bacteria**	**Strictly fungi**	**Bacteria and fungi (mixed)**	
Note	Clean _(35)_	8 (3.8)	2 (0.9)	24 (11.3)	34 (16.0)
	Dirty _(110)_	38 (17.8)	5 (2.3)	63 (29.6)	106 (49.8)
	**Total **_**(145)**_	**46 (21.6)**	**7 (3.3)**	**87 (40.8)**	**140 (65.7)**
Coin	Clean _(30)_	13 (6.1)	4 (1.8)	10 (4.6)	27 (12.6)
	Dirty _(38)_	14 (6.5)	5 (2.3)	14 (6.5)	33 (15.5)
	**Total **_**(68)**_	**27 (12.6)**	**9 (4.2)**	**24 (11.2)**	**60 (28.2)**
**Grand total **_**(213)**_		**73 (34.3)**	**16 (7.5)**	**111 (52.1)**	**200 (93.9)**

N = Number obtained, Notes: Bacteria: χ^2^-test: χ^2^ = 13.29; df = 1; P < 0.001; Fungi: χ^2^-test: χ^2^ = 0.00; df = 1; P = 0.991, Bacteria and fungi: χ^2^-test: χ^2^ = 12.42; df = 1; P < 0.001.

Coins: Bacteria: χ^2^-test: χ^2^ = 1.37; df = 1; P = 0.242; Fungi: χ^2^-test: χ^2^ = 0.003; df = 1; P = 0.959; Bacteria and fungi: χ^2^-test: χ^2^ = 13.06; df = 1; P < 0.001.

All samples (100%) collected from butchers and hospitals were contaminated (Table 3). Overall, there was a significant difference (χ^2^ = 131.857, df = 6, P<0.001) in contamination with respect to source of currency. The highest bacterial contamination (13.6%) was detected in samples from hospitals while mixed contamination was highest (15.0%) in samples from food vendors (Table 3). Contamination with only bacteria (χ^2^ = 80.824, df = 5, P<0.001), only fungi (χ^2^ = 30.357, df = 5, P<0.001) and both bacteria and fungi (χ^2^ = 97.371, df = 5, P<0.001) was a significant (Table 3).

**Table 3:** Relationship between source of currency and type of contamination

**Source (N)**	**Contamination N (%)**	**Total contaminated**	**Bacteria and fungi (mixed)**	**N (%)**
	**Strictly bacteria**	**Strictly fungi**		
Butchers (35)	8 (3.8)	1 (0.5)	26 (12.2)	35 (100)
Food vendors (56)	17 (7.9)	3 (1.4)	32 (15.0)	52 (92.9)
Store attendants (19)	7 (3.3)	2 (0.9)	8 (3.8)	17 (77.3)
Students (22)	7 (3.3)	2 (0.9)	12 (5.6)	21 (93.5)
Hospital/Clinic (60)	29 (13.6)	4 (1.9)	27 (12.6)	29 (100)
Taxi drivers (21)	5 (2.3)	4 (1.9)	6 (2.8)	15 (71.4)
**Total (213)**	**73 (34.2)**	**16 (7.5)**	**111 (52.1)**	**200 (93.9)**

N = Number obtained; Bacteria: χ^2^-test: χ^2^ = 80.824; df = 5; P < 0.001; Fungi: χ^2^-test: χ^2^ = 30.357; df = 5; P < 0.001. Bacteria and fungi: χ^2^-test: χ^2^ = 97.371; df = 5; P < 0.001.

Nine bacterial species (Table 4) and 15 fungal species (Table 5) were isolated. Among the bacteria isolated, coagulase negative *Staphylococcus* (CoNS) (54.9%) and *Staphylococcus aureus* (20.1%) were the predominant isolates. These isolates recorded the highest frequency of occurrence on all notes and coins except 500 frs coin where *Bacillus sp* (3, 1.4%) recorded the highest frequency of occurrence. *P. mirabilis* (0.4%) was the least isolated (Table 4). There was no significant difference observed in the distribution of bacteria isolates between currency denominations (Table 4).

**Table 4:** Distribution of bacteria isolated with respect to currency type, denomination and source

**Currency denomination**		**Bacteria isolated N (%)**								
		***Bacillus sp***	***Corynebacterium sp***	***Pseudomonas sp***	***Staphylococcus aureus***	**CoNS**	***Shigella dysenteriae***	***Escherichia coli***	***Proteus mirabilis***	***Enterobacter aerogenes***
Note	500	6 (2.8)	0 (0.0)	1 (0.5)	3 (1.4)	13 (6.1)	0 (0.0)	2 (0.9)	0 (0.0)	0 (0.0)
	1000	7 (3.2)	10 (4.7)	4 (1.8)	10 (4.7)	29 (10.7)	2 (0.9)	4 (1.8)	1 (0.5)	0 (0.0)
	2000	3 (1.4)	2 (0.9)	7 (3.2)	12 (5.6)	28 (13.1)	1 (0.5)	1 (0.5)	0 (0.0)	2 (0.9)
	5000	4 (1.8)	1 (0.5)	1 (0.5)	2 (0.9)	9 (4.2)	1 (0.5)	2 (0.9)	0 (0.0)	0 (0.0)
	10000	1 (0.5)	1 (0.5)	2 (0.9)	5 (2.3)	6 (2.8)	1 (0.5)	3 (1.4)	0 (0.0)	0 (0.0)
Coin	50	4 (1.8)	1 (0.5)	1 (0.5)	5 (2.3)	10 (4.7)	0 (0.0)	3 (1.4)	0 (0.0)	0 (0.0)
	100	1 (0.5)	2 (0.9)	2 (0.9)	5 (2.3)	20 (9.4)	1 (0.5)	2 (0.9)	0 (0.0)	0 (0.0)
	500	3 (1.4)	1 (0.5)	0 (0.0)	1 (0.5)	2 (0.9)	1 (0.5)	1 (0.5)	0 (0.0)	0 (0.0)
**Total**		**29 (13.6)**	**18 (8.4)**	**18 (8.4)**	**43 (20.1)**	**117 (54.9)**	**7 (3.3)**	**18 (8.4)**	**1 (0.4)**	**2 (0.9)**
**χ**^**2**^**-test**		**χ**^**2**^** = 13.512df = 7 P = 0.061.**	**χ**^**2**^** = 13.610df = 7 P = 0.059**	**χ**^**2**^** = 6.132df = 7P = 0.524**	**χ**^**2**^** = 5.714df = 7P = 0.574.**	**χ**^**2**^** = 9.873 df = 7 P = 0.196**	**χ**^**2**^** = 4.063 df = 7 P = 0.773.**	**χ**^**2**^** = 5.806 df = 7 P = 0.563.**	**χ**^**2**^** = 3.50 df = 7 P = 0.835.**	**χ**^**2**^** = 8.027 df = 7 P = 0.330.**
Source of currency										
Butchers		2 (0.9)	8 (3.7)	5 (2.3)	11 (5.1)	23 (10.7)	1 (0.5)	2 (0.9)	0 (0.0)	1 (0.5)
Food vendors		6 (2.8)	8 (3.7)	4 (1.8)	9 (4.2)	32 (15.0)	1 (0.5)	3 (1.4)	1 (0.5)	0 (0.0)
Store attendants		1 (0.5)	2 (0.9)	1 (0.5)	3 (1.4)	10 (4.7)	0 (0.0)	4 1.8)	0 (0.0)	1 (0.5)
Students		5 (2.3)	0 (0.0)	2 (0.9)	2 (0.9)	7 (3.2)	1 (0.5)	5 (2.3)	0 (0.0)	0 (0.0)
Hospitals/Clinic		12 (5.6)	0 (0.0)	5 (2.3)	12 (5.6)	39 (18.3)	3 (1.4)	4 (1.8)	0 (0.0)	0 (0.0)
Taxi drivers		3 (1.3)	0 (0.0)	1 (0.5)	6 (2.8)	6 (2.8)	1 (0.5)	0 (0.0)	0 (0.0)	0 (0.0)
**Total**		**29 (13.6)**	**18 (8.4)**	**18 (8.4)**	**43 (20.2)**	**117 (54.9)**	**7 (3.2)**	**18 (8.4)**	**1 (0.5)**	**2 (0.9)**
**χ**^**2**^**-test**		**χ**^**2**^** = 9.966 df = 7 P = 0.126.**	**χ**^**2**^** = 24.399 df = 7 . P < 0.001**	**χ**^**2**^** = 3.920 df = 6 P = 0.688.**	**χ**^**2**^** = 10.485 df = 6 P = 0.106.**	**χ**^**2**^** = 32.725 df = 6 P < 0.001.**	**χ**^**2**^** = 2.549 df = 6 P = 0.863.**	**χ**^**2**^** = 15.261 df = 6 P = 0.018.**	**χ**^**2**^** = 3.103 df = 6 P = 0.7**	**χ**^**2**^** = 7.362 df = 6 P = 0.289.**

N = Number isolated.

**Table 5:** Distribution of fungal isolates with respect to currency type, denomination and source

**Currency denomination**		**Fungi isolated N (%)**														
		***Absidia sp***	***Acremonium sp***	***Aspergillus sp***	***Blastomyces sp***	***Epidermophyton sp***	***Fusrium sp***	***Scopulariopsis sp***	***Sporothrix sp***	***Microsporum sp***	***Candida albicans***	***Candida sp***	***Penicillium sp***	***Rhyzopus sp***	***Saccharomyces cerevisiae***	***Trichophyton sp***
Note	500	*0 (0.0)*	0 (0.0)	5 (2.3)	0 (0.0)	0 (0.0)	0 (0.0)	0(0.0)	0 (0.0)	0 (0.0)	0 (0.0)	2 (0.9)	4 (1.8)	3 1.4)	1(0.5)	2 (0.9)
	1000	0 (0.0)	0 (0.0)	11 (5.1)	2 (0.9)	2 (0.9)	0 (0.0)	0 (0.0)	2(0.9)	2 (0.9)	2 (0.9)	4 (1.8)	11 (5.1)	5 (2.3)	8 (3.7)	6 (2.8)
	2000	1 (0.5)	0 (0.0)	10 (4.7)	0 (0.0)	0 (0.0)	0 (0.0)	1 (0.5)	0 (0.0)	0 (0.0)	2 (0.9)	6 (2.8)	10 (4.6)	5 (2.3)	4 (1.8)	3 (1.4)
	5000	0 (0.0)	0 (0.0)	3 (1.4)	0 (0.0)	0 (0.0)	0 (0.0)	0 (0.0)	0 (0.0)	0 (0.0)	0 (0.0)	1 (0.5)	0 (0.0)	1 (0.5)	0 (0.0)	1 (0.5)
	10000	0 (0.0)	1 (0.5)	1 (0.5)	0 (0.0)	0 (0.0)	0 (0.0)	0 (0.0)	0 (0.0)	1 (0.5)	1 (0.5)	1 (0.5)	0 (0.0)	**3 (1.4**)	**3 (1.4)**	0 (0.0)
Coin	50	0 (0.0)	0 (0.0)	2 (0.9)	0 (0.0)	1 (0.5)	0 (0.0)	0 (0.0)	0 (0.0)	0 (0.0)	0 (0.0)	1 (0.9)	3 (1.4)	0 (0.0)	1 (0.5)	0 (0.0)
	100	0 (0.0)	0 (0.0)	2 (0.9)	0 (0.0)	1 (0.5)	1 (0.5)	0 (0.0)	0 (0.0)	0 (0.0)	0 (0.0)	4 (1.8)	5 (2.3)	2 (0.9)	**6 (2.8)**	5 (2.3)
	500	0 (0.0)	0 (0.0)	3 (1.4)	0 (0.0)	1 (0.5)	0 (0.0)	0 (0.0)	0 (0.0)	0 (0.0)	2 (0.9)	0 (0.0)	1 (0.5)	0 (0.0)	0 (0.0)	1 (0.0)
**Total**		**1 (0.5)**	**1 (0.5)**	**37 (17.3)**	**2 (0.9)**	**5 (2.3)**	**1 (0.5)**	**1 (0.5)**	**2 (0.9)**	**3 (1.4)**	**7 (3.2)**	**19 (8.9)**	**34 (15.9)**	**19 (8.9)**	**23 (10.7)**	**18 (8.4)**
**χ**^**2**^**-test**		**χ**^**2**^ **= 3.996 df = 7 P = 0.780**	**χ**^**2**^ **= 12.525 df = 7 P = 0.085**	**χ**^**2**^ **= 9.490 df = 7 P = 0.219**	**χ**^**2**^ **= 7.042 df = 7 P = 0.425**	**χ**^**2**^ **= 5.969 df = 7 P = 0.543**	**χ**^**2**^ **= 4.893 df = 7 P = 0.673**	**χ**^**2 **^**=3.996 df = 7 P = 0.780**	**χ**^**2 **^**=7.042 df = 7 P = 0.425**	**χ**^**2**^ **= 7.576 df = 7 P = 0.371**	**χ**^**2**^ **= 12.617 df = 7 P = 0.082**	**χ2 = 3.367 df = 7 P = 0.849**	**χ**^**2**^ **= 10.061 df = 7 P = 0.185**	**χ**^**2**^ **= 6.875 df = 7 P = 0.442**	**χ**^**2**^ **= 9.170 df = 7 P = 0.241**	**χ**^**2**^ **= 6.132 df = 7 P = 0.524**
**Source of currency**																
Butchers		1 (0.5)	0 (0.0)	8 (3.7)	0 (0.0)	2 (0.9)	0 (0.0)	0 (0.0)	2 (0.9)	2 (0.9)	0 (0.0)	5 (2.3)	2 (0.9)	5 (2.3)	4 (1.8)	6 (2.8)
Food vendors		0 (0.0)	0 (0.0)	12 (5.6)	2 (0.9)	1 (0.5)	1 (0.5)	1 (0.5)	0 (0.0)	1 (0.5)	3 (1.4)	4 (1.8)	12 (5.6)	5 (2.3)	8 (3.7)	8 (3.7)
Store attendants		0 (0.0)	0 (0.0)	1 (0.5)	0 (0.0)	0 (0.0)	0 (0.0)	0 (0.0)	0 (0.0)	0 (0.0)	0 (0.0)	1 (0.5)	4 (1.8)	4 (1.8)	1 (0.5)	1 (0.5)
Students		0 (0.0)	1 (0.5)	7 (3.2)	0 (0.0)	0 (0.0)	0 (0.0)	0 (0.0)	0 (0.0)	0 (0.0)	0 (0.0)	1 (0.5)	10 (4.7)	0 (0.0)	2 (0.9)	0 (0.0)
Hospital/Clinic		0 (0.0)	0 (0.0)	5 (2.3)	0 (0.0)	2 (0.9)	0 (0.0)	0 (0.0)	0 (0.0)	0 (0.0)	3 (1.4)	5 (2.3)	6 (2.8)	5 (2.3)	8 (3.7)	2 (0.9)
Taxi drivers		0 (0.0)	0 (0.0)	4 (1.8)	0 (0.0)	0 (0.0)	0 (0.0)	0 (0.0)	0 (0.0)	0 (0.0)	1 (0.5)	3 (1.4)	0 (0.0)	0 (0.0)	0 (0.0)	1 (0.5)
Butchers		1 (0.5)	0 (0.0)	8 (3.7)	0 (0.0)	2 (0.9)	0 (0.0)	0 (0.0)	2 (0.9)	2 (0.9)	0 (0.0)	5 (2.3)	2 (0.9)	5 (2.3)	4 (1.8)	6 (2.8)
Food vendors		0 (0.0)	0 (0.0)	12 (5.6)	2 (0.9)	1 (0.5)	1 (0.5)	1 (0.5)	0 (0.0)	1 (0.5)	3 (1.4)	4 (1.8)	12 (5.6)	5 (2.3)	8 (3.7)	8 (3.7)
**χ**^**2**^**-test**		**χ**^**2**^ **= 5.567 df = 5 P = 0.473**	**χ**^**2**^ **= 9.450 df = 5 P = 0.150**	**χ**^**2**^ **= 13.880 df = 5 P = 0.031**	**χ**^**2**^ **= 6.233 df = 5 P = 0.398**	**χ**^**2**^ **= 4.197 df = 5 P = 0.650**	**χ**^**2**^ **= 3.103 df = 5P = 0.796**	**χ**^**2**^ **= 3.103 df = 5 P = 0.796**	**χ**^**2**^ **= 11.183 df = 5 P = 0.083**	**χ**^**2**^ **= 7.181 df = 5 =0.304**	**χ**^**2**^ **= 4.872 df = 5 P = 0.560.**	**χ**^**2**^ **= 4.822 df = 5 P = 0.567.**	**χ**^**2**^ **= 28.674 df = 5 P < 0.001**	**χ**^**2**^ **= 11.081df = 5 P = 0.086**	**χ**^**2**^ **= 6.542** **df = 5** **P = 0.365**	**χ**^**2**^ **= 12.751** **df = 5** **P = 0.04**

N = Number isolated.

All bacteria isolates (except *P. mirabilis* and *E. aerogenes*) were recovered on samples from butchers and food vendors.

Samples from store attendants also comprised another major source of isolates as all bacteria species except *P. mirabilis* and *Shigella dysenteriae* were recovered from this source. The highest occurrence of bacterial species was found in the hospital samples. The distribution of *Corynebacterium* (χ^2^ = 24.39, df = 5, P < 0.001), CoNS (χ^2^ = 32.72, df = 5, P < 0.001) and *E. coli* (P = 0.018) on currency from the various sources was statistically significant (Table 4).

Of the fungal species isolated, *Aspergillus sp* (17.3%) and *Penicillium sp* (15.9%) had the highest frequency of occurrence (Table 5). With the exception of the 10,000 frs notes and the 100 frs coin whose predominant fungal isolates were *Rhyzopus sp* (3, 1.4%) and *Saccharomyces* (6, 2.8%) respectively, these two species had the highest recovery rate from all currency denominations. There was no significant difference observed in the distribution of these isolates between currency denominations (Table 5). *Aspergillus sp* and *C. albicans* were present in currency from all sources. With the exception of *Absidia* and *Acremonium sp*, all fungal species isolated were detected in currency from food vendors. The distribution of *Aspergillus sp* (χ^2^ = 13.880, df = 5, P = 0.031), *Penicillium sp* (χ^2^ = 28.674, df = 5, P < 0.001) and *Trichophyton sp* (χ^2^ = 12.751, df = 5, P = 0.047) with respect to source of currency was statistically significant (Table 5).

Only *Proteus mirabilis* and *Shigella dysenteriae* were sensitive (100%) to all antibiotics (Table 6). Isolates showed varying susceptibility to β-lactam antibiotics. Generally low susecptbilities to penicillins was observed whereas susceptibility to cephalosporins was higher. Susceptibility to the penicillins tested was 7.3%, 32% and 36.3% respectively to amoxicillin, penicillin G and ampicillin respectively. Isolates also showed low susceptibility to cotrimoxazole (39.5%). The most effective antibiotics were cefuroxime, gentamicin, norfloxacin and oxacillin as all isolates (100%) were susceptible to these agents (Table 6). *Staphylococcus aureus*, one of our predominant isolates was completely resistant (100%) to amoxicillin, penicillin and vancomycin. Coagulase-negative *Staphylococcus* was also resistant (100%) to vancomycin. *Pseudomonas aeruginosa* was resistant (100%) to amoxicillin and ampicillin but some strains showed resistance to cefotaxime, ceftriaxone and cotrimoxazole (Table 6).

**Table 6:** Susceptibility (%) of bacteria isolates to antibiotics

**Antibiotic (% susceptible)**														
**Isolate (N)**	**AML**	**AMP**	**CTX**	**CRO**	**CXM**	**C**	**SXT**	**E**	**CN**	**NOR**	**OX**	**P**	**TE**	**VA**
*Bacillus sp* (29)	0(0.0)	0(0.0)	29(100)	29(100)	29(100)	29(100)	29(100)	29(100)	29(100)	29(100)	29(100)	17(58.6)	29(100)	29(100)
*Corynebacterium sp* (18)	0(0.0)	18(100)	18(100)	18(100)	18(100)	18(100)	0(0.0)	18(100)	18(100)	18(100)	18(100)	18(100)	18(100)	18(100)
*Pseudomonas aeruginosa* (18)	0(0.0)	0(0.0)	4(22.2)	8(44.4)	18(100)	18(100)	6(33.3)	18(100)	18(100)	18(100)	18(100)	18(100)	18(100)	18(100)
*Staphylococcus aureus* (43)	0(0.0)	43(100)	43(100)	43(100)	43(100)	43(100)	43(100)	8(18.6)	43(100)	43(100)	43(100)	0(0.0)	10(23)	0(0.0)
*Shigella dysenteriae* (07)	7(100)	7(100)	7(100)	7(100)	7(100)	7(100)	7(100)	7(100)	7(100)	7(100)	7(100)	7(100)	7(100)	7(100)
*Escherichia coli* (18)	0(0.0)	0(0.0)	18(100)	14(77.7)	18(100)	14(77.7)	12(66.7)	18(100)	18(100)	18(100)	18(100)	18(100)	18(100)	18(100)
CONS	0(0.0)	21(17.9)	117(100)	117(100)	117(100)	117(100)	0(0.0)	117(100)	117(100)	117(100)	117(100)	0(0.0)	52(44.4)	0(0.0)
*Proteus mirabilis* (01)	1(100)	1(100)	1(100)	1(100)	1(100)	1(100)	1(100)	1(100)	1(100)	1(100)	1(100)	1(100)	1(100)	1(100)
*Enterobacter aerogenes* (02)	2(100)	2(100)	2(100)	2(100)	2(100)	2(100)	2(100)	2(100)	2(100)	2(100)	2(100)	2(100)	0(0.0)	2(100)
**Total (253)**	**10(7.3)**	**92(36.3)**	**239(94.4)**	**239(94.4)**	**253(100)**	**249(98.4)**	**100(39.5)**	**218(86.1)**	**253(100)**	**253(100)**	**253(100)**	**81(32.0)**	**153(60)**	**93(36.7)**

N = number tested;% = percentage susceptible; ampicillin (AMP), amoxicillin (AML), cefotaxime (CTX), ceftriaxone (CRO), cefuroxime (CXM), chloramphenicol (C), cloxacillin (CX), cotrimoxazole (SXT), erythromycin (E), gentamicin (CN), oxacillin (OX), penicillin G (P), tetracycline (TE) and vancomycin (VA). CONS: Coagulase Negative *Staphylococcus.*

## Discussion

We analyzed a total 213 samples of currency comprising 145 notes and 68 coins of various denominations, physical conditions and from different sources (Additional file 1). Two hundred (93.9%) samples were contaminated with different bacterial and fungal species. Majority of samples were contaminated with both bacteria and fungi (mixed contamination) than with only bacteria or fungi (Tables 1, 2 and 3). The isolation of both bacteria and fungi from currency as demonstrated by our study shows that currency could play an important role in the transmission of microbial agents in the community and thus presents a public health threat. Contamination of currency in our study is higher than reported in currencies in other developing countries like Nigeria, 52.5% [15], Nepal, 75% [26] and Saudi Arabia 72.3% [27] but comparable to the 94% reported in the United States [12] and lower than 100% recently reported in Ghana [20] and Pakistan [28]. These differences reflect differences in hygienic practices and handling of currency in different areas and also show that microbial contamination of currency is a global problem. Several behavioural practices in our study site may contribute to currency contamination: keeping money under body surfaces, improper washing of hands after using the toilet, wetting fingers with saliva when counting currency, coughing and sneezing on hands and handling currency, placement or storage of money on dirty surfaces during transactions and spraying during ceremonies. Generally notes (140/145, 96.6%) showed higher contamination than coins (60/68, 88.2%) (Tables 1 and 2). Paper currency (notes) has a cotton/linen composition and offers a large surface as breeding ground for microbes which can persist on it for longer periods. In addition, the surface of banknotes is not smooth, but irregular facilitating adherence by many different types of microbes. These factors ease colonization of notes more than coins. Coins are metallic in nature and depending upon the composition, they have been shown to possess antimicrobial activity. CFA (XAF) coins are nickel based. Coins composed of nickel alloy (nickel-brass and copper-nickel) to be more inhibitory to microbial colonization than coins of other metals [29]. In addition, the smaller surface area of coins provides a smaller surface for microbial colonization. These factors may account for our observation. Microbial growth was not detected in 16 new currency (‘mint’) samples used as control. It could be that these samples were contaminated by fastidious organisms and the media and/or culture conditions employed were inappropriate for their isolation. Since this money had not been in circulation we suggest usage and handling, as the possible causes of contamination of Franc CFA (XAF) in circulation.

The extent of contamination was found to be related to currency denomination (Table 1). There was a significant relationship in currency denomination and contamination (χ^2^ = 3.868, df = 1, P = 0.049) with lower denominations showing higher levels of contamination. Our findings are consistent with recent reports [20,28,30,31]. Lower denominations pass through more hands for different daily transactions than higher denominations. The higher rate of exchange predisposes lower denominations to higher levels of contamination. Also, the tendency to mishandle currency is more among lower denominations than currency of higher values. Contrary to the results of Sabahat and Humaira [28] which did not suggest any one currency to be particularly susceptible to contamination, we observed the highest level of contamination (100%) among the 500 frs notes (Table 1). Our findings indicate that lower denominations harbor the greatest bulk of infectious agents particularly as they are exchanged more than higher denominations. However, no denomination was protected from contamination as we detected microbial growth in all denominations of notes and coins analyzed.

We also observed contamination of currency to be associated with the physical state of currency and source (Table 2 and 3). Contamination solely with bacteria (χ^2^ = 13.29, df = 1, P<0.001) and with both bacteria and fungi (χ^2^ = 12.42, df = 1, P<0.001) was significantly higher in dirty notes than in clean notes (Table 2) indicating a direct relationship between physical condition of the currency and contamination. Physical appearance of currency could serve as an indication of age of currency. Age has been suggested as another important factor that determines the occurrence of microorganisms on currency [32,33].

All currency (100%) obtained from butchers and the hospital/clinic was contaminated. Highest contamination with both bacteria and fungi was observed in samples from food vendors (Table 3). Food either cooked or uncooked may contain microorganisms which can be transferred directly or indirectly through currency. Without proper hygiene practices, microorganisms can be transferred from currency to food. Thus, simultaneous handling of food and currency, a common practice among food vendors in study area could transfer pathogenic microorganisms to food. Handling of money between hand washing and food handling requires a repeat of the process of hand washing before handling of food. The detection of microbial contamination in currency from all sources is a great concern and indicates that contaminated currency could pose a health threat particularly to immune compromised individuals both in the hospital and in the community. Improved personal hygiene standards are highly solicited to reduce risk of infection from currency.

Pathogenic and potential bacterial species isolated from notes and coins included coagulase negative *Staphylococcus* (CoNS) (54.9%), *Staphylococcus aureus* (20.1%), *Bacillus sp* (13.6%), *Corynebacterium sp* (8.4%), *Pseudomonas aeruginosa* (8.4%), *Escherichia coli* (8.4%)*, Shigella dysenteriae* (3.3%), *Enterobacter aerogenes* (0.9%) and *Proteus mirabilis* (0.4%) (Table 4). Our findings show that gram positive organisms are the predominant contaminants of CFA (XAF) circulating in study area. However, the isolation of both gram positive and gram-negative bacteria indicates that it could be serving as a reservoir of pathogenic organisms. There was no significant difference in the distribution of bacteria isolates on notes and coins of various denominations (Table 4). Coagulase-negative *Staphylococcus, Staphylococcus aureus, E. coli* and *Bacillus sp* were detected in all denominations of notes and coins.

The staphylococci (coagulase negative and *S. aureus*) were the predominant isolates in currency from all sources (Table 4), an indication of their ubiquitous nature. These organisms are normal flora of the skin and mucous membranes. Coagulase-negative staphylococci have long been regarded as non-pathogenic but their important role as pathogens and their increasing incidence have been recognized and studied in recent years. *S. aureus* are well-recognized pathogens [34]. Kumar *et al.*[21] have shown that *S. aureus* can survive on paper notes for eight days. Prolonged survival of this pathogen on currency permits transmission.

*Bacillus species* are spore-forming organisms that inhabit the soil and are ubiquitous in the environment. The isolation of *Bacillus species* from currency shows contamination with soil material. *Bacillus species* are generally perceived to be inconsequential. However previous studies [35] have highlighted the relevance of other *Bacillus species* as etiologic agents of non-gastrointestinal infections.

*Corynebacteriun sp* and *Pseudomonas aeruginosa* were other potentially pathogenic organisms recovered in currency. *Pseudomonas aeruginosa* is an important opportunistic pathogen causing a wide range of acute and chronic infections when introduced into areas devoid of normal defences [36].

Enteric pathogens *E. coli* and *S. dysenteriae* as well as potential pathogens *P. mirabilis* and *E. aerogenges* were isolated from samples analyzed. *E. coli* and *S. dysenteriae* were widely distributed in various denominations of currency (Table 4) than the other organisms. Their detection in currency is indicative of fecal contamination and poor sanitary conditions of the environment and personal hygiene practices of currency handlers. Thus, simultaneous handling of money and food should be discouraged unless proper hygiene is observed or food handling tools are used between the two processes. Our findings suggest CFA (XAF) as a vehicle for transmission of human pathogenic bacteria as money is in constant circulation.

Although *E. coli* is generally regarded as an opportunistic pathogen, studies [10,18] have isolated the pathogenic strain O157:H7 from currency. This strain of *E. coli* organism has been shown to survive in currency for up to eleven days [10] making possible its transfer to humans. Sharma and Dhanashree [37] investigated the RAPD profiles of pathogens isolated from notes and coins and reported similar banding patterns of some isolates obtained from these sources confirming spread of pathogens through currency.

Fifteen species of fungi (Table 5) were isolated from both notes and coins from all sources. *Aspergillus species* predominated. *Aspergillus species* produce ochratoxins and aflatoxins. Aflatoxins have been shown to be carcinogenic [38]. In addition, inhalation of spores of *their spores* may cause aspergillosis making this organism a health threat. *Penicillium* can cause pneumonia [39]. *Rhizopus* is an agent for zygomycosis and eye infections [40]. *Candida albicans*, though a normal flora of humans causes a secondary infection in HIV/AIDS patients [41]. Its isolation from currency in a locality with high prevalence of HIV (as in other countries in the Central African sub-region) may present a challenge to the prevention of candidiasis in HIV infected individuals.

Bacteria isolated were generally susceptible to most of the antibiotics tested. Ceftriaxone, gentamicin, norfloxacin and ofloxacin were the most active drugs as they were effective against all isolates (100%) (Table 6). Other effective drugs included erythromycin (86.1%), cefotaxime (94.4%), cefuroxime (94.4%), and chloramphenicol (98.4%). Inactive drugs were amoxicillin, penicillin, ampicillin, cotrimoxazole and vancomycin as they showed low susceptibilities of 7.3%, 32%, 36.3%, 39.5% and 36.7% respectively. Resistant strains were found mostly among *Pseudomonas aeruginosa, E. coli, Staphylococcus aureus* and coagulase-negative *Staphylococcus. Staphylococcus aureus* and coagulase negative *Staphylococcus* were vancomycin resistant. Our findings have far-reaching public health implications because the CFA (XAF) is used in other countries in the Central African sub-region. Previous reports [42,43] show that multidrug resistant *Staphylococccus aureus* and *P. aeruginosa* are widely distributed in study area. Our findings further confirm the ubiquity of drug resistant organisms in study environment with currency serving as a possible route for transmission. Since the CFA (XAF) franc is also used in other countries, there is the possibility of spread of these resistant pathogens to other countries, or it could be that these have been introduced from these countries. This implies that spread of these pathogens could be transboundary. To the best of our knowledge, this is the first study that has investigated the CFA (XAF) as a possible route for transmission of pathogenic organisms. Findings call for further investigation of the CFA (XAF) in various countries in the CEMAC zone to generate evidence-based findings for the urgent need for national and regional policies on currency handling and use. Also, molecular studies establishing a link between isolates from currency and those of clinical origin need to be carried out to ascertain the role of currency in transmission of pathogens.

### Limitations

We did not include a control strain in antibiotic susceptibility testing. Also, the MIC of active drugs was not determined. Methods used to isolate microorganisms detected only culturable organisms. Molecular methods could have detected non-culturable or fastidious organisms. In addition, we did not investigate for other microorganisms such as protozoa or viruses which are likely to be common causes of gastrointestinal or respiratory illnesses in the community.

## Conclusions

Our study has shown that currency circulating in Buea District could facilitate the spread of resistant microorganisms. We therefore recommend that behavioural practices that could predispose to infection from currency or currency contamination should be discouraged and rigorous sensitization campaigns on health risks of poor hygiene during or after handling of currency carried out. To minimise the use of cash and hence the possibility of exposure to contaminated currency either electronic credit cards or plastic (or polymer-based) CFA (XAF) which could be easily washed to reduce contamination as found in other countries could be instituted for all transactions. Mutilated and worn-out money should be withdrawn frequently from circulation as they are the most contaminated. Policies on proper handling of currency should be introduced to preserve their quality and longevity.

## Abbreviations

Franc CFA: Franc de la Communauté Financiere d’Afrique; XAF: ISO Currency Code for the Central African CFA franc; BEAC: Bank of Central African States (Banque des Etats de l`Afrique Central); CEMAC: Central African Economic and Monetary Community; FSA: Food Science Australia; BICEC: Banque Internationale du Cameroun pour l’Eparnge et le Credit.

## Competing interests

The authors have declared that they have no competing interests

## Authors’ contributions

JTKA conceived, designed and coordinated the study, interpreted data and initiated the writing of the manuscript. GN collected samples, isolated and characterized bacteria carried out antimicrobial susceptibility testing. HMD participated in isolation and characterization of bacteria. TKN together with JTKA conceived, designed and coordinated the study, and approved the final version of the manuscript. All authors read and approved the final manuscript.

## Supplementary Material

Additional file 1 **Distribution of currency denominations by physical appearance.** Description of data: This table shows the distribution of various denominations of currency analyzed with respect to type (note or coin) and physical appearance (clean or dirty). Click here for file
